# Dynamics of Metabolite Induction in Fungal Co-cultures by Metabolomics at Both Volatile and Non-volatile Levels

**DOI:** 10.3389/fmicb.2018.00072

**Published:** 2018-02-05

**Authors:** Antonio Azzollini, Lorenzo Boggia, Julien Boccard, Barbara Sgorbini, Nicole Lecoultre, Pierre-Marie Allard, Patrizia Rubiolo, Serge Rudaz, Katia Gindro, Carlo Bicchi, Jean-Luc Wolfender

**Affiliations:** ^1^School of Pharmaceutical Sciences (EPGL), University of Geneva, University of Lausanne, Centre Médical Universitaire (CMU), Geneva, Switzerland; ^2^Department of Drug Science and Technology, University of Turin, Turin, Italy; ^3^Plant Protection, Mycology and Biotechnology, Agroscope, Nyon, Switzerland

**Keywords:** fungal co-culture, metabolomics, induced metabolites, antifungal, volatile, non-volatile, dynamics

## Abstract

Fungal co-cultivation has emerged as a promising way for activating cryptic biosynthetic pathways and discovering novel antimicrobial metabolites. For the success of such studies, a key element remains the development of standardized co-cultivation methods compatible with high-throughput analytical procedures. To efficiently highlight induction processes, it is crucial to acquire a holistic view of intermicrobial communication at the molecular level. To tackle this issue, a strategy was developed based on the miniaturization of fungal cultures that allows for a concomitant survey of induction phenomena in volatile and non-volatile metabolomes. Fungi were directly grown in vials, and each sample was profiled by head space solid phase microextraction gas chromatography mass spectrometry (HS-SPME-GC-MS), while the corresponding solid culture medium was analyzed by liquid chromatography high resolution mass spectrometry (LC-HRMS) after solvent extraction. This strategy was implemented for the screening of volatile and non-volatile metabolite inductions in an ecologically relevant fungal co-culture of *Eutypa lata* (Pers.) Tul. & C. Tul. (Diatrypaceae) and *Botryosphaeria obtusa* (Schwein.) Shoemaker (Botryosphaeriaceae), two wood-decaying fungi interacting in the context of esca disease of grapevine. For a comprehensive evaluation of the results, a multivariate data analysis combining Analysis of Variance and Partial Least Squares approaches, namely AMOPLS, was used to explore the complex LC-HRMS and GC-MS datasets and highlight dynamically induced compounds. A time-series study was carried out over 9 days, showing characteristic metabolite induction patterns in both volatile and non-volatile dimensions. Relevant links between the dynamics of expression of specific metabolite production were observed. In addition, the antifungal activity of 2-nonanone, a metabolite incrementally produced over time in the volatile fraction, was assessed against *Eutypa lata* and *Botryosphaeria obtusa* in an adapted bioassay set for volatile compounds. This compound has shown antifungal activity on both fungi and was found to be co-expressed with a known antifungal compound, *O*-methylmellein, induced in solid media. This strategy could help elucidate microbial inter- and intra-species cross-talk at various levels. Moreover, it supports the study of concerted defense/communication mechanisms for efficiently identifying original antimicrobials.

## Introduction

It is well established that microbial natural products (NPs) represent one of the most important resources for the discovery of potent antimicrobials (Koehn and Carter, 2005; Newman and Cragg, 2016).

Finding new antimicrobial agents remains key in fighting pathogens for both human health and agronomical needs, particularly to address the challenge related to resistance phenomena. Fungal species represent an abundant source of bioactive metabolites with very high chemodiversity. Approximately 42% of microbial bioactive molecules have been produced by fungi, and many of these compounds have been widely used as antifungals and antibiotics (Demain, 2014; Schüffler and Anke, 2014). The biosynthetic potentials of such microorganisms are, however, challenging to study in a comprehensive manner, as it is known that their defense pathways are inducible (Gomez-Escribano and Bibb, 2011). To better characterize their metabolome and discover novel bioactive molecules, strategies eliciting pathways controlled by silent genes have to be implemented.

In this respect, the biosynthesis of novel metabolites has been successfully induced by the OSMAC approach (Bode et al., 2002; Pérez Hemphill et al., 2017) and by using epigenetic modifiers (Chen et al., 2013; Chung et al., 2013; Du et al., 2014). Microbial strains have also been modified by mutagenesis or genetic engineering for this purpose (Bergmann et al., 2007).

Unlike the above mentioned approaches, microorganism co-culture is a strategy inspired by nature, involving inter-microbial competition and communication for the induction of new metabolites through the activation of silent genes (Netzker et al., 2015). This methodology is crucial for drug discovery because it allows, often in combination with metabolomic approaches (Marmann et al., 2014; Wu et al., 2015), for the identification of new compounds. Furthermore, many elicited metabolites show interesting biological activities (e.g., antibacterial/antifungal). In this respect, fungal co-cultivation has emerged as a powerful strategy for the induction of new bioactive fungal metabolites (Bertrand et al., 2014b). To date, various co-cultivation strategies have been applied focusing on synthetic biology (Goers et al., 2014) and secondary metabolite production (Bertrand et al., 2014b; Netzker et al., 2015).

Recent studies have been conducted with the aim of identifying non-volatile agar diffused induced compounds in fungal co-cultures by using different metabolite profiling methods: liquid chromatography mass spectrometry (LC-MS), LC-ultraviolet (UV), and nuclear magnetic resonance (NMR). For example, a combination of UV- and NMR-based techniques was used to highlight the production of induced secondary metabolites by a co-culture of sponge-associated Actinomycetes (Dashti et al., 2014). Other studies have been conducted to analyze single cultures and co-cultures using LC-MS-based metabolomic approaches (Adnani et al., 2015; Bohni et al., 2016). With such approaches, MS-guided isolation of highlighted biomarkers can be performed efficiently for full *de novo* identification of the metabolites of interest (Azzollini et al., 2016).

Compared with differential LC-MS-based profiling studies, the identification of volatile induced compounds in fungal co-cultures by gas chromatography mass spectrometry (GC-MS) approaches remains an emerging and less explored field (Bertrand et al., 2014b). For example, the use of headspace solid-phase microextraction and headspace sorptive extraction was successfully applied in the detection of volatile metabolites produced by toxigenic *Fusarium* species (Demyttenaere et al., 2004). Moreover, volatile biomarkers in *Pseudomonas aeruginosa* and *Aspergillus fumigatus* mono- and co-cultures were identified by analyzing headspace samples using thermal desorption coupled with GC-MS (Neerincx et al., 2016).

Such studies have highlighted the complexity and high variability of induction phenomena in fungal co-cultures. Historically, *in vitro* fungal co-cultures were obtained on solid media, as fungi grow well in these conditions and are prompt to colonize unexplored regions with nutrients (Prosser and Tough, 1991). This approach has allowed for the study of interaction patterns and metabolic changes that take place at the mycelial front (Boddy, 2000; Woodward and Boddy, 2008; Bertrand et al., 2014b).

Solid medium co-cultures are usually cultivated in Petri dishes measuring 9 or 15 cm in diameter. Extraction and sample preparation for further metabolite profiling represent a significantly time-consuming step that requires large amounts of solvent at such a large scale for analytical purposes only. We previously showed that a miniaturization of co-culture experiments on solid media is well adapted for acquiring fast metabolite fingerprints of large number of replicates needed for time-series metabolomic investigations. To this end, a procedure based on multi-well 2 cm Petri dishes was developed (Bertrand et al., 2014a).

Considering all the methodological advancements made in the field, the survey of metabolite variations in both volatile and non-volatile fractions may become of great interest for a comprehensive investigation of the induction phenomena that are likely to occur in microbial co-cultures. Thus, in this study, we developed a miniaturized procedure for a concomitant analysis of the volatile and non-volatile fractions of the same microbial co-culture by metabolomics in time-series experiments.

Such studies involve several experimental factors and give rise to complex datasets with multiple sources of variability. Dedicated chemometric tools have been developed for this purpose. Among them, the ANOVA Multiblock OPLS method (AMOPLS) was reported as an efficient approach for the assessment of the specific impact of controlled experimental factors (Boccard and Rudaz, 2016). The method allows for an evaluation of the main effects of the factors, as well as their interactions, with a single model based on specific scores and loadings.

In this work, a strategy was developed and applied to reveal the induction of volatile and non-volatile metabolites over time in an ecologically relevant fungal co-culture of two wood-decaying fungi interacting in the context of esca disease of grapevine (*Eutypa lata* and *Botryosphaeria obtusa*) (Glauser et al., 2009; Hofstetter et al., 2012). This strategy allowed for the evaluation of both the developed procedure and the related data-mining approach. It further revealed the dynamics of induction of volatile and non-volatile metabolites and allowed for the assessment of their antimicrobial activities.

## Materials and Methods

### Chemicals

The extractions were performed using methanol (HPLC grade, Fisher Scientific) and dichloromethane (HPLC grade, VWR International). The UHPLC-Orbitrap MS analyses were performed using ULC/MS-grade acetonitrile and a mixture of water and formic acid (FA) purchased from Biosolve. Potato Dextrose Agar, (PDA, Difco) was used as the culture medium.

The standard compounds 2-nonanone, decane, and undecane were purchased from Sigma–Aldrich.

### Biological Material

Two wood-decaying fungi involved in esca disease were selected as a model for this work. The *Eutypa lata* and *Botryosphaeria obtusa* were isolated from the experimental vineyards of ACW Changins (Switzerland). The two fungal strains were stored at 4°C in the Agroscope ACW bank, in vials containing a diluted potato dextrose broth solution (1:4)^1^.

### Experimental Settings of Culture and Co-culture Conditions

*Eutypa lata* and *Botryosphaeria obtusa* single cultures were prepared by placing 2 mm agar plugs of fungal pre-cultures in the center of the vial. The co-cultures were prepared by placing two 2 mm agar plugs of a pre-culture of the two different fungal species on the opposite sides of the SPME vial. Immediately after the inoculation, each vial was closed with an appropriate SPME cap (HDSP cap 18 mm magnetic PTFE/Sil, Agilent Technologies, United States). Blank samples (only PDA agar) were prepared. The cultures were incubated at 21°C in the dark.

For optimization, 2 and 4 mL of PDA agar were placed in a SPME vial. Three replicates of each single culture and co-culture for both volume conditions were analyzed, after 7 days of growth, by GC-MS. A 2 mL volume of PDA was selected for further time-point experiments.

For the study of the evolution of volatile and non-volatile metabolite induction over time, five replicates (*n* = 5) of each single culture and co-culture were analyzed by GC-MS and LC-MS at 2, 4, 7, and 9 days after incubation. One blank sample (only agar) was also analyzed at each time point, yielding a total of 4 blanks.

### HS-SPME-GC-MS for the Analysis of the Volatile Fractions

The headspace of each individual single culture and co-culture was sampled with a 2 cm DVB/CAR/PDMS SPME fiber (Supelco, Bellefonte, PA, United States) directly in the SPME vial used for cultivation. Sampling was carried out by an MPS2 autosampler (Gerstel, Mülheim a/d Ruhr, Germany) at 40°C (no pre-equilibrium, 30 min of sampling). The fiber was automatically injected in an Agilent 7890 GC coupled to an Agilent 5975C MS (Agilent, Little Falls, DE, United States). Sampling was only performed once for each culture to avoid any perturbation of the volatile fraction over time. The GC-MS conditions were as follows: inlet temperature 250°C, split injection (5 min, 1/10 split ratio), carrier gas (helium at a flow rate of 1 mL/min); column: HP5MS (30 m × 0.25 mm i.d. × 0.25 μm; Agilent Technologies). Volatile metabolite profiling was carried out with the following temperature program: 50°C (1 min) – 3°C/min – 200°C – 15°C/min – 250°C (4 min). MS was operated in the EI mode at 70 eV with a mass range of 35–350 amu in full-scan mode.

### GC-MS Data Processing and Deconvolution of GC Peaks

Data were processed with Agilent MSD ChemStation version D.03.00.611 and Shimadzu GCMSsolution version 2.51. GC peaks were identified by comparing their linear retention indices (calculated *versus* a C9–C25 hydrocarbon mixture) and their MS by comparison with those present in commercially available libraries (Wiley, Adams, NIST).

### Extraction of the Non-volatile Fraction for LC-HRMS

Each fungal pure strain culture, co-culture and non-inoculated agar sample (blank) was freeze-dried (Freeze Dryer Alpha 2–4 LD plus, Martin Christ Gefriertrocknungsanlagen GmbH). Nine milliliters of the extraction solvent mixture (dichloromethane–methanol 64:36) were added to each vial. The extraction was performed for 20 min by sonication directly in the vials in a water-bath sonicator (Ultrasonic Cleaver 5200, Branson Ultrasonics Corporation). The sonicated samples were filtered through glass cotton. Finally, the extracts were dried under vacuum using a centrifugal evaporator (Genevac HT-4, SP scientific, Ipswich, Suffolk, United Kingdom). Each extract was weighed.

### UHPLC-Orbitrap-MS Analysis

Before analysis, solid-phase extraction (SPE), using a Sep Pak Vac SPE C18 cartridge (1 cc, 100 mg, Waters, Milford, MA, United States), was performed to remove highly non-polar compounds that would be incompatible with reverse phase chromatography. Briefly, the extract was dissolved in methanol-water at 7 mg/mL and eluted using methanol-water 95:5 (v/v) through the C18 cartridge; the fraction was then dried and weighed. Finally, each sample was redissolved in MeOH 100% at 4 mg/mL to be analyzed by HRMS. Chromatographic separation was performed on a Thermo Dionex Ultimate 3000 UHPLC system interfaced with a Q-Exactive Plus mass spectrometer (Thermo Scientific, Bremen, Germany), using a heated electrospray ionization (HESI-II) source in both positive- and negative-ion modes. The LC conditions were as follows: column, Waters BEH C18 50 × 1.0 mm i.d., 1.7 μm; mobile phase, (A) water with 0.1% formic acid and (B) acetonitrile with 0.1% formic acid; flow rate, 300 μL/min; injection volume, 1 μL; initial isocratic step at 5% of B for 0.2 min, followed by a linear gradient of 5-100% of B over 4 min, and finally an isocratic step at 100% of B for 3.50 min. Diisooctyl phthalate C24H38O4[M + H]^+^ ion (*m/z* 391.28429) was used as an internal lock mass. The optimized HESI-II parameters were as follows: source voltage, 3.5 kV (pos), 3.3 kV (neg); sheath gas flow rate (N_2_), 48 units; auxiliary gas flow rate, 11 units; spare gas flow rate, 2; capillary temperature, 300°C; and S-Lens RF Level, 55. The mass analyzer was calibrated using a mixture of caffeine, methionine, arginine, phenylalanine, alanine, and acetate.

### LC-MS Data Processing and Peak Picking

ThermoRAW data were converted to the open MS format (.mzXML) using ProteoWizard. Data treatment and the dereplication process were performed using MZmine 2.14.2. The Dictionary of Natural Products was used as the database for the dereplication^2^. The data treatment steps were performed using MZmine 2.14.2. Features detected from blank samples were removed from the generated matrix. The entire procedure for the feature detection is reported in Supplementary Table S2. This procedure allowed for the generation of a list of features (*m/z* @RT *×* peak area).

### Multivariate Data Analysis (AMOPLS)

ANOVA Multiblock Orthogonal Partial Least Squares (AMOPLS) was computed under the MATLAB^®^ 8 environment (The MathWorks, Natick, MA, United States) with combinations of OPLS routines implemented in the KOPLS toolbox (Bylesjö et al., 2008) and in-house functions. The starting point of the AMOPLS analysis takes advantage of the experimental design to partition the experimental matrix (X) into a series of additive submatrices (X_i_) based on average values related to each of the factors levels. By these means, the relative variability of each main effect or interaction term can be assessed by computing the sum of squares of the submatrices. The latter are then analyzed as a multiblock structure using an extension of the OPLS model. Similarly to classical multivariate analysis (e.g., Principal Component Analysis), observation groupings can be highlighted using scores, while variable contributions are associated with loading values. As it is based on the OPLS framework, the AMOPLS model separates predictive latent variables that are related to the experimental factors with associated score (tp) and loading vectors (pp) from orthogonal components summarizing the unexplained variability (scores to and loadings po). Level barycenters of the experimental factors are used as a response matrix, and specific AMOPLS components can be easily associated with the different effects based on the contribution of each submatrix (X_i_) to a given component. The statistical significance of each effect is evaluated using Monte Carlo random permutations of the experimental design, and an effect-to-residuals ratio is used to compute empirical *p*-values. A series of 10^4^ random permutations was performed to validate the AMOPLS model and assess the statistical significance of the main and interaction effects. For further details, the reader can refer to the original article describing the AMOPLS method (Boccard and Rudaz, 2016).

### Bioassay

To evaluate the antifungal activity of the volatile induced compounds, a 9 cm Petri dish, divided in two by a plastic septum, was used. This partition allowed only for the diffusion of volatile molecules between the two sectors of the dish. Pure cultures of *E. lata* and *B. obtusa* were inoculated on PDA in the first sector of the Petri dish, using a 2 mm agar plug of a fungal pre-culture. The volatile compound was injected into 5 mm filter paper posed on a glass disk in the second sector of the Petri dish. In a preliminary antifungal assay, the activity of 2-nonanone, decane, and undecane (Sigma–Aldrich) was assessed at a concentration of 774 μL/L. The concentrations reported for this bio-assay are expressed as the liquid volume of each volatile compound on filter paper per dish volume (for simplification, the dish volume was calculated without considering the plastic septum) (Neri et al., 2007). To further assess the antifungal potency of 2-nonanone, a bioassay was used to test five concentrations of 2-nonanone: 154.8 μL/L, 77.4 μL/L, 19.3 μL/L, 9.7 μL/L, and 2.4 μL/L. Mycelia growth was monitored on days 3, 5, 7, and 9. For each concentration, six replicate experiments were performed.

## Results and Discussion

### Concomitant Volatile/Non-volatile Metabolite Profiling and Integrated Mining of Metabolomic Data

A strategy based on the cultivation of fungal single cultures and co-cultures in solid phase microextraction (SPME) vial was developed to study the evolution of volatile and non-volatile metabolite induction at four different time points (2, 4, 7, and 9 days) in a fungal co-culture of *Eutypa lata* and *Botryosphaeria obtusa.*

Co-cultivation in a 20 mL SPME vial was chosen, as this system allows for the exploration of the volatile and non-volatile metabolome in a miniaturized format permitting high throughput screening, while generating a sufficient number of replicates. Moreover, because the diameter of the well and the diameter of the SPME vial are 2 cm in both cases, this format matches our previously developed 12-well-plate-cultivation system. Cultivation in such a miniaturized system was found to accelerate the production of metabolites by several weeks compared with cultivation in commonly used 9 cm Petri dishes (Bertrand et al., 2014a).

The two fungal species in the co-culture were inoculated at the same time in the SPME vial to capture the signaling molecular events related to the onset of the interaction. Co-inoculation was also found to be necessary to avoid the loss of volatile metabolites and the perturbation of the volatile fraction composition due to SPME vial opening. In parallel, single cultures were grown under the same conditions for comparison.

Before running the whole time-series study, a smaller set of experiments was carried out to guarantee an adequate solid media volume for metabolite production. Three single cultures and co-cultures were grown in 2 and 4 mL Potato Dextrose Agar (PDA) media. The evaluation of the metabolites’ profiles indicated that increasing the medium volume did not significantly influence the richness of the volatile metabolome or its replicate variability (Supplementary Figure S1).

To study the dynamics of metabolite induction in the co-culture, five replicates for each single culture and co-culture at four time points (2, 4, 7, and 9 days) were simultaneously prepared. Each sample was left to grow in the dark, mimicking the natural growth conditions of these fungi in the wood of *Vitis vinifera* L., and was analyzed by headspace (HS)-SPME-GC-MS. The HS SPME sampling of the atmosphere surrounding the living microorganisms allowed for efficient automated GC-MS profiling of all mono- and co-cultures.

To maintain the same sampling timing for the analysis of both volatile and non-volatile metabolomes, each culture sample was frozen at -80°C immediately after the GC-MS analysis to further quench biochemical reactions. The solid media samples were lyophilized and extracted by a dichloromethane-methanol solvent mixture for subsequent liquid chromatography high resolution mass spectrometry (LC-HRMS) analysis of the non-volatile fraction.

For the GC-MS analysis, direct desorption of the SPME fiber provided a comprehensive view of the metabolites sampled from the volatile fraction of both single cultures and co-cultures. The GC-EI-MS dataset could not be directly exported for peak picking and subsequent data mining because of extensive fragmentation in the electron impact (EI) mode and the presence of non-discriminant main fragments. To convert the GC-EI-MS dataset into features that could be mined, all chromatographic peaks detected in GC-MS chromatograms were integrated and the corresponding features (RT *×* peak area) exported. The corresponding EI-MS spectra of all features were examined carefully to align all chromatographic peaks precisely. Based on the abovementioned data treatment, putative structures were assigned to most of the features by comparing mass spectra and their linear retention indices (LRI) (calculated versus a C9–C25 hydrocarbon mixture) with those present in commercially available libraries (Wiley, Adams, NIST). This comparison allowed for the generation of a detailed and informative GC-MS matrix of all compounds (Cpds ID @ RT @ LRI *×* samples *×* peak area), referred to as “GC-MS features” in the data-mining section of this paper.

In parallel, LC-MS fingerprints were acquired on the fungal samples obtained after lyophilization and solvent extraction. The extracts were dried under vacuum and then subjected to reversed-phase solid-phase extraction (SPE) to remove highly non-polar compounds and reduce carryover effects in LC-MS. The samples were finally analyzed using UHPLC-Orbitrap-HRMS. This procedure takes advantage of the high-resolution and rapid separation capabilities of UHPLC as well as the high-mass accuracy offered by HRMS for metabolite annotation. The LC-MS data matrix was automatically obtained as follows: all LC-MS features (*m/z @*RT *×* peak area) detected in the fingerprints of each replicate were deconvoluted using an automated optimized peak picking procedure for the retrieval of the relevant chromatographic ion traces (Pluskal et al., 2010).

To obtain a comprehensive view of the metabolic alterations due to *Culture* and *Time* conditions, a multivariate approach was carried out that allows for the decomposition of different parameters of the experimental design and their correlation to the evolution profile. To this end, AMOPLS was independently applied to both GC-MS and LC-MS datasets.

**FIGURE 1:**
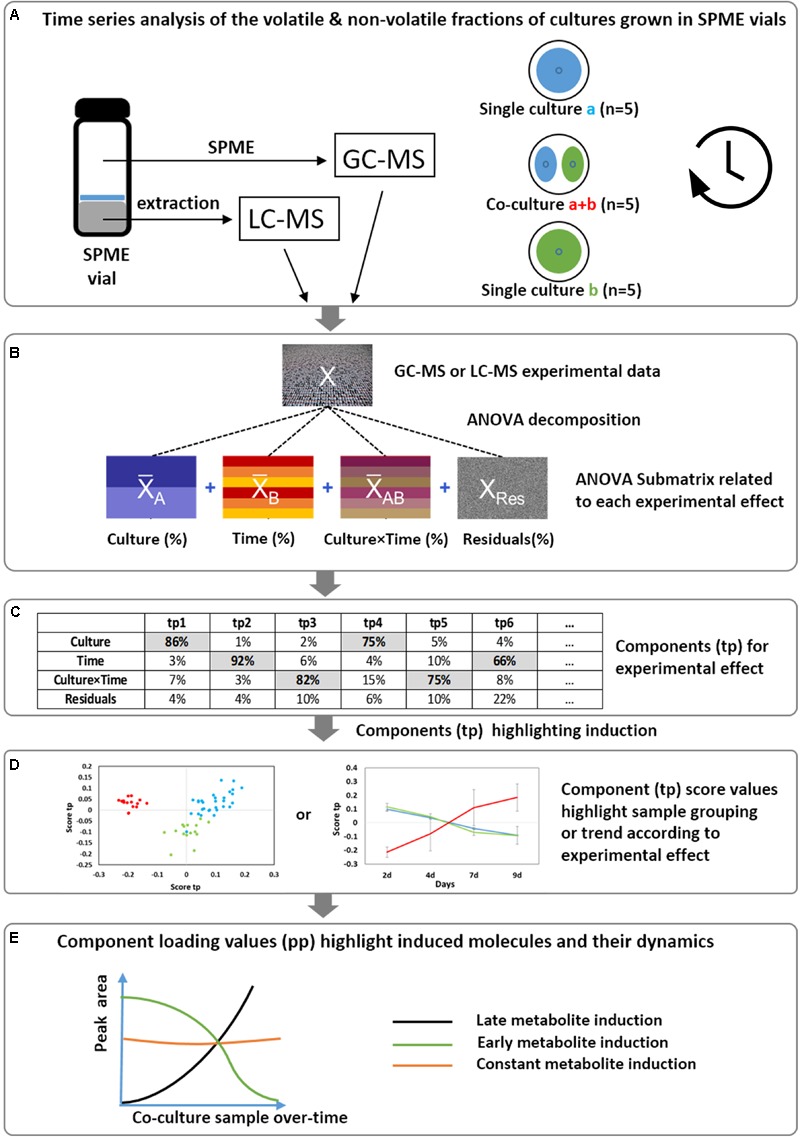
Overview of the implemented strategy to reveal the induction of volatile and non-volatile molecules and highlight their dynamics. **(A)** Fungal single cultures and co-cultures are grown directly in SPME vials and analyzed at different time points. The volatile fraction is directly analyzed by head-space GC-MS, while the whole culture medium is profiled by LC-HRMS after solvent extraction. Two different and independent datasets are obtained. **(B)** An AMOPLS model is applied separately to GC-MS and LC-MS datasets starting with the decomposition of the matrix (X) in a series of submatrices (X_i_) according to the experimental design *(Culture, Time, Culture × Time)*. **(C)** The relative variability of each main effect can be assessed by computing the sum of squares of the submatrices. **(D)** The submatrices are analyzed using a multiblock OPLS model. AMOPLS separates the sources of variability related to the experimental factors with dedicated predictive score (tp) and loading values (pp), from the orthogonal components summarizing unexplained variations. **(E)** The components loading values highlight induced molecules with respect to the different experimental factors.

The AMOPLS workflow (**Figure 1**) starts with the ANOVA decomposition of the experimental matrix (X) into a series of submatrices (X_i_) according to the experimental design (*Culture, Time* and their potential interaction). By computing the sum of squares of each X_i_ table, the proportion of total variability associated with each effect was evaluated and compared in a quantitative manner. A joint analysis of the latent structures related to these submatrices was then carried out using a multiblock Orthogonal Partial Least Square model. The AMOPLS model allowed for the different effects of the experimental design to be efficiently distinguished. The co-cultivation *(Culture* main effect) was associated with constitutive metabolic differences due to culture conditions (single cultures vs. co-culture) shared over all incubation times (i.e., 2, 4, 7, and 9 days). The effect related to the evolution of the metabolome composition over time (with *Time* as the main effect) was related to dynamic metabolic signatures observed for all culture conditions (i.e., common for single cultures and co-cultures). Finally, the *Culture × Time* interaction was linked to dynamic metabolic inductions due to the interaction between microorganisms according to the incubation time.

Based on the model block weights, distinct predictive AMOPLS components could be related to each effect and specifically investigated to highlight relevant trends in sample distributions. The loadings related to these components allowed for the contribution of each variable to be evaluated and highlighted induced molecules with respect to different experimental factors.

### Detection of Induced Metabolites and Their Dynamics

The AMOPLS model was applied to both GC-MS and LC-MS datasets separately. Induction phenomena in the volatile fraction were highlighted in the deconvoluted GC-MS dataset after unit variance scaling.

**FIGURE 2:**
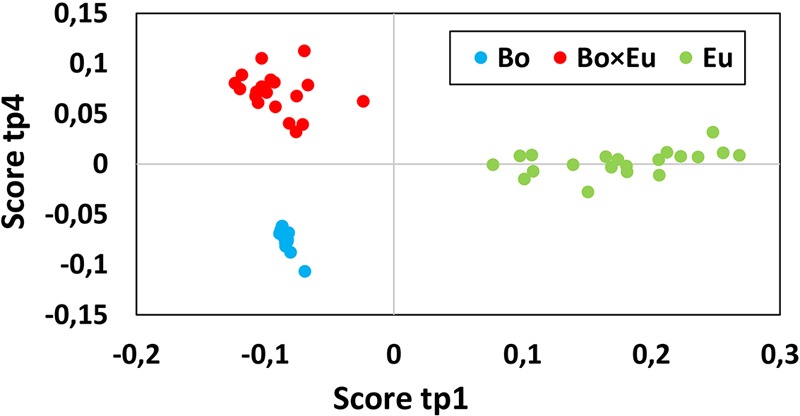
The score plot of the predictive component tp1 (horizontal axis) and tp4 (vertical axis) associated with the *Culture* effect for the volatile dataset. Clusters related to single cultures (blue dots for *B. obtusa* and green dots for *E. lata*) and co-cultures (red dots) are clearly separated. Calculated variance is 25.1% for tp1 and 12% for tp4. Co-culture samples present a negative score value on tp1 and a positive score value on tp4.

The total volatile data matrix (Cpds ID @ RT @ LRI *×* peak area) was decomposed by ANOVA into a series of submatrices. The relative variance associated with each effect was evaluated using the sum of squares of each table as follows: *Culture* 37.1%, *Time* 10.3%, interaction *Culture × Time* 11.9% and Residuals 40.7%. These results clearly underlined the marked impact of the culture conditions on the measured volatile metabolites. Block contributions were computed to relate AMOPLS predictive components (tp) to specific effects of the experimental design (*Culture, Time* or *Culture × Time*), as in Supplementary Table S1a. The score plot of the predictive components associated with the *Culture* effect was investigated to highlight relevant groupings to distinguish single cultures from co-cultures. The AMOPLS predictive components, tp1 and tp4, were found to be associated with the *Culture* effect and distinguished single cultures from co-cultures (**Figure 2**).

Co-culture samples presented a negative score value on the component tp1 and a positive score value on the component tp4 (red dots in **Figure 2**). The corresponding GC-MS features with negative loading values on pp1 and positive loading values on pp4 were induced in the co-culture. Based on those pp1 and pp4 loading values, features induced in the co-culture were highlighted, as presented in **Table 1**. Features were ranked based on pp4 and further filtered by keeping only the features with negative pp1 values, leading to a subset of the most extreme values.

**Table 1:** Features induced in the co-culture highlighted by a positive loading value on pp4 and a negative loading value on pp1 related to the corresponding score plot in **Figure 2** (red dots).

Compound	RT	LRI	pp1	pp4
RT: 6.704	6.704	901	-0.00081833	0.01224223
Decane	11.198	1002	-0.00140068	0.0109626
p-Mentha-1(7),8-diene	10.638	1009	-0.00086617	0.0108786
Decane, 2-methyl-	12.43	1051	-0.00154911	0.01047015
RT:11.611	11.611	1032	-0.00160635	0.01044977
RT:13.361	13.361	1073	-0.00160741	0.01032619
1-Nonen-3-ol	12.857	1062	-0.0016277	0.01017094
2-Bornene	7.789	909	-0.00163079	0.01016206
Undecane	15.517	1104	-0.00160385	0.01010671
2-Nonanone	14.159	1092	-0.0005042	0.01009972
RT:9.322	9.322	974	-0.00088581	0.00977868
2-Heptanone	6.403	892	-0.00077665	0.00955368
sesqui@RT34.879	34.879	1590	-0.00078607	0.00952052
RT:8.377	8.377	947	-0.00107459	0.00916112
RT:26.017	26.017	1366	-0.00118651	0.00823336
RT:12.647	12.647	1057	-0.00144774	0.00728384

Most of the induced features (**Table 1**) were tentatively annotated based on their mass spectra and linear retention index compared with those present in commercially available databases and, when possible, through co-injection of the corresponding commercial standards. The non-annotated features were labeled by the retention times and linear retention index of their associated GC chromatographic peaks according to the similarity of their corresponding GC-EI-MS spectra.

**FIGURE 3:**
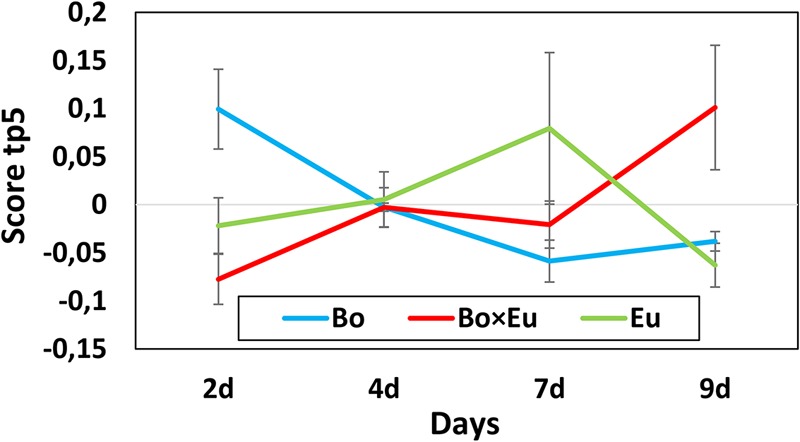
Scores of predictive component tp5 associated with the *Culture × Time* effect for the volatile dataset. The score value is reported on the vertical axis while the days of growth of the fungal cultures are indicated on the horizontal axis. A clear differentiation between single cultures (blue line = *B. obtusa*; green line = *E. lata*) and co-cultures (red line) could be observed on day 2 and day 9.

**FIGURE 4:**
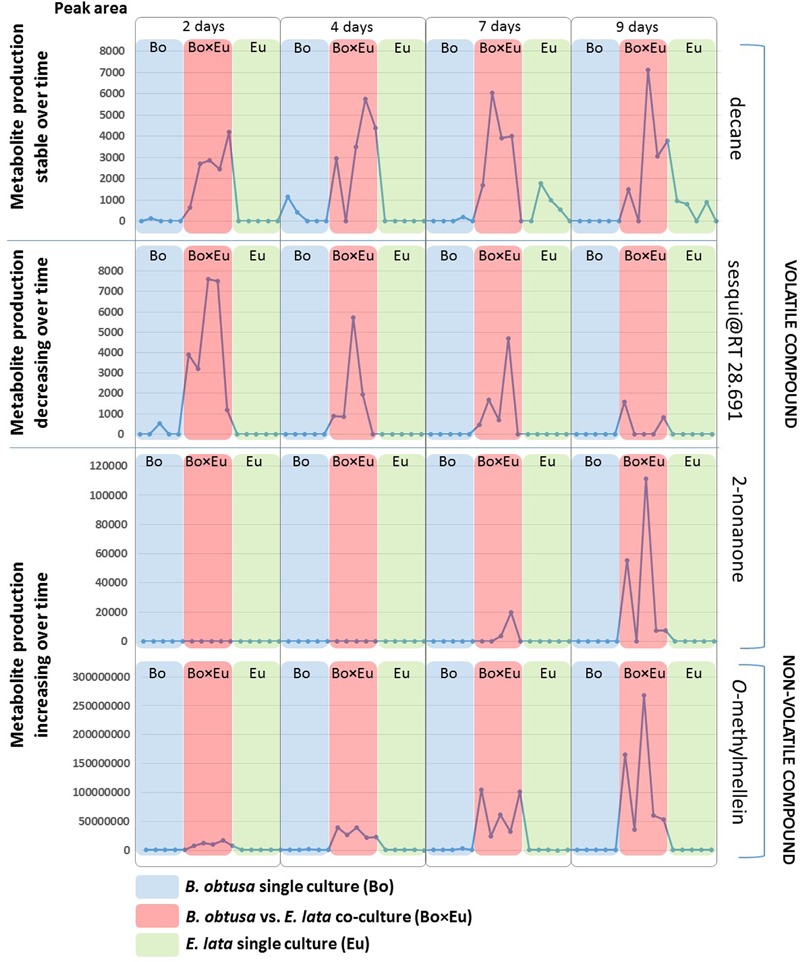
Induction patterns of volatile and non-volatile compounds detected in the fungal co-cultures. Each dot represents a replicate of fungal single cultures or co-cultures. Red boxes indicate co-culture (Bo × Eu), while blue boxes indicate the *B. obtusa* single culture (Bo) and green boxes *E. lata* single cultures (Eu). The peak areas in both GC-MS and LC-MS (vertical axis) of the detected compounds is reported for each sample (horizontal axis) at different time points (2, 4, 7, and 9 days). Most of the induced volatile compounds were detected over a long time span as shown for the case of decane in the first section of the figure. Early molecule production, decreasing over-time (as in second section of the figure) was observed for a volatile sesquiterpenoid compound (sesqui@RT 34.879). The third section of the figure illustrates compounds increasingly produced in the co-culture over time. This is the case of the volatile 2-nonanone and of the non-volatile *O*-methylmellein.

The dynamics of metabolite induction were further revealed by in-depth investigation of the *Culture × Time* effects. Accordingly, the score plot of the predictive components associated with the *Culture × Time* effect was analyzed. In this case, the tp3 versus (vs.) tp5 score plot did not allow for the detection of clear groupings; however, the score was evaluated independently on tp3 and tp5. The predictive component tp5 was selected because of its clear trend of continuous induction over time (**Figure 3** red trace for co-culture). The associated GC-MS features with positive pp5 loading values were thus induced over time in the co-culture. These features were 2-nonanone (**Figure 4**) and an unidentified sesquiterpenoid with a molecular weight of 222 Da @ RT of 34.879 (LRI: 1590). The identity of 2-nonanone was confirmed through the co-injection of a commercially available standard (Supplementary Figure S4), while for the sesquiterpenoid, only the compound type could be attributed based on the fragmentation pattern of its associated EI-MS (Supplementary Figure S2). This GC-MS feature is referred to as sesqui@RT 34.879.

The induction of 2-nonanone and the sesqui@RT 34.879 was incremental over time despite a high variability of metabolite induction. This phenomenon is well known in microbial interactions (Bertrand et al., 2014b) and justifies the use of replicates for highlighting induction phenomena.

Conversely, negative loading values of GC-MS features showed decreasing abundances over time. In this case, only a single minor unidentified sesquiterpene with a molecular weight of 204 Da @ RT of 28.691 (LRI: 1431) was characterized by this induction pattern (**Figure 4**). Also in this case, the compound type could be attributed based on the fragmentation pattern of its associated EI-MS (Supplementary Figure S3). This GC-MS feature is referred to as sesqui@RT 28.691.

The other volatile compounds (**Table 1**) present in the co-culture and absent, or hardly detectable, in the monoculture were characterized by a rather constant level at all timings in the confrontation experiment, e.g., decane (**Figure 4**).

Generally, volatile organic compounds (VOCs), characterized by a high vapor pressure and a low molecular weight, play a significant role in the communication between microorganisms and act as infochemicals (Santoro et al., 2011; Effmert et al., 2012). In particular, the detected VOC 2-nonanone incrementally produced over time in the co-culture is known to be produced by bacteria such as *Pseudomonas* sp. (Popova et al., 2014; Briard et al., 2016) and by fungi (Zhao et al., 2017). This compound presents antifungal activity against *Fusarium* sp. (Raza et al., 2015), as well as against *Penicillum* sp. (Neri et al., 2006). Moreover, the compound was recently reported to be active against gastrointestinal nematodes (Ortu et al., 2017). Its detection in this confrontation model confirms the potential of our comprehensive approach for detecting induced VOCs in fungal interactions. The evaluation of the compound’s biological activity was further studied as described in Subheading “Antifungal Activity of the Induced Compounds” of the Results and Discussion.

**FIGURE 5:**
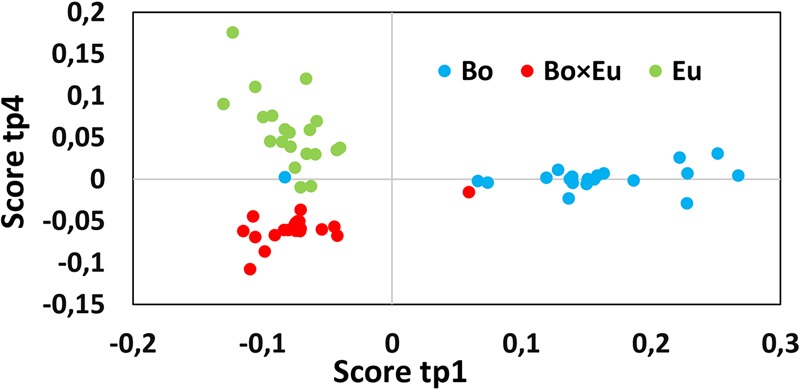
The score plot of the predictive component, tp1 (horizontal axis) and tp4 (vertical axis) associated with the *Culture* effect for the non-volatile dataset. Clusters related to single cultures (blue dots for *B. obtusa* and green dots for *E. lata*) and co-cultures (red dots) are separated. Calculated variance is 11.2% for tp1 and 5% for tp4. Co-culture samples present a negative score value on both tp1 and tp4.

Induction phenomena in the non-volatile fraction of the co-culture were highlighted following a data-mining strategy similar to that for GC-MS. In this case, the data matrix was composed of all the non-volatile features detected in positive ionization (*m/z @RT ×* peak area), measured in single cultures and co-cultures at four different time points. The AMOPLS model was established based on this non-volatile dataset after unit variance scaling to identify the induction of low-abundance biomarkers. The non-volatile data matrix was decomposed by ANOVA, and the relative variance associated with each effect was evaluated using the sum of squares of each submatrix as follows: *Culture* conditions 16.2%, *Time* 13.8%, interaction *Culture × Time* 15.3%, and Residuals 54.7%. Block contributions were computed to relate AMOPLS predictive components to specific effects of the experimental design (*Culture, Time* or *Culture × Time*), as in Supplementary Table S1b. For the LC-MS dataset, the score plots of the predictive components linked to the *Culture* effect were investigated to highlight relevant groupings to distinguish single cultures from co-cultures. The AMOPLS predictive components, tp1 and tp4, in relation to the *Culture* main effect highlighted relevant groupings to distinguish single cultures and co-cultures in the tp1–tp4 score plot (**Figure 5**). In this case, however, no *m/z* features characterized by negative loading values on both pp1 and pp4 could be clearly associated and highlighted. This result suggests that the compounds that were present only in the co-culture and were rather stable over time were not detected.

**FIGURE 6:**
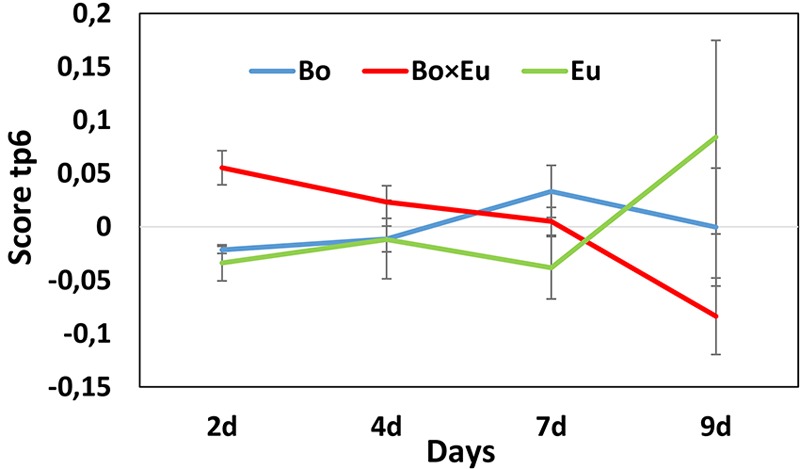
Scores of the predictive component tp6 are associated with the *Culture × Time* effect for the non-volatile dataset. The score value is reported on the vertical axis, while days of growth of the fungal cultures are shown on the horizontal axis. A clear differentiation between single cultures (blue line = *B. obtusa*; green line = *E. lata*) and co-cultures (red line) could be observed on day 9.

To reveal the metabolites that can be induced over time, the *Culture × Time* effect was taken into consideration. Accordingly, the predictive components related to the *Culture × Time* factor (tp3, tp5, tp6) were evaluated to highlight relevant groupings at different time points. No clear clustering between single cultures and co-cultures was obtained, and subsequently, the score was evaluated independently on tp3, tp5, and tp6. The predictive component tp6 was selected for further investigation because of its significant trend of continuous induction over time (red trace in **Figure 6**). The associated *m/z* features with negative pp6 loading values were thus found to be induced over time in the co-culture. For this induction pattern, the feature *m/z* 193.09@RT1.44 was highlighted (pp6 = -6.78740623). As for the volatile induced molecules, a large variability of intensities of metabolite induction was observed (**Figure 4**). This compound was dereplicated based on HRMS measurement as *O*-methylmellein, based on its molecular formula and its occurrence in *E. lata.* As shown in **Figure 4**, *O*-methylmellein increased over time from day 2 to day 9 [Supplementary Figure S5 shows the extracted ion chromatogram (XIC) of *O*-methylmellein detected at day 9 in two single-culture samples and one co-culture sample].

This induced metabolite was previously described in the confrontation zone excised, using a razor blade, from a whole 9 cm co-culture of *E. lata* vs. *B. obtusa* (Glauser et al., 2009). The identification of this confrontation marker further validated the pertinence of the proposed miniaturized strategy for the detection of non-volatile induced compounds and the interest of investigating the induction pattern dynamics.

Interestingly, the production of volatile mellein-related compounds by fungi has been previously described (Jelén and Grabarkiewicz-Szczȩsna, 2005) they were, however, not detected in the volatile fraction analyzed by SPME-GC-MS.

The application of the AMOPLS model to the negative ionization LC-MS dataset did not highlight any induced feature in the co-culture.

### Antifungal Activity of the Induced Compounds

To better understand the possible role of the volatile and non-volatile induced compounds identified in the confrontation, their biological activities were investigated. *O*-methylmellein was previously reported to have antifungal activity against *B. obtusa* (Glauser et al., 2009). Similarly, the possible antimicrobial activity of the identified volatile compounds, and that could be purchased as standards, was assessed against both fungi involved in the ecological confrontation. Indeed, induced compounds could present an antimicrobial activity as part of the communication interplay between these *Vitis vinifera* wood-decaying fungi.

To this end, a bioassay that mimics the effects of the volatile induced compounds on fungi growing *in vitro* on solid media was adapted based on previous study (Briard et al., 2016). The principle of the assay was to monitor the growth of the fungus, inoculated in the lid of a small Petri dish and enclosed in a large Petri dish in the presence of a volatile compound soaked into a paper filter inside a small plastic cup in the larger Petri dish. In our case, a standard 9 cm Petri dish divided in two by a septum, allowing for the diffusion of the volatile compound, was used for the assay. The method was found simple to implement as there was no need to use the smaller, additional Petri dish that needed to be sterilized first (which, in turn, would increase the overall duration of the test). In the first sector of the Petri dish, the fungus was inoculated; in the other sector, the volatile compound was injected into the filter paper at different doses, allowing for the saturation of the Petri dish’s entire volume.

**FIGURE 7:**
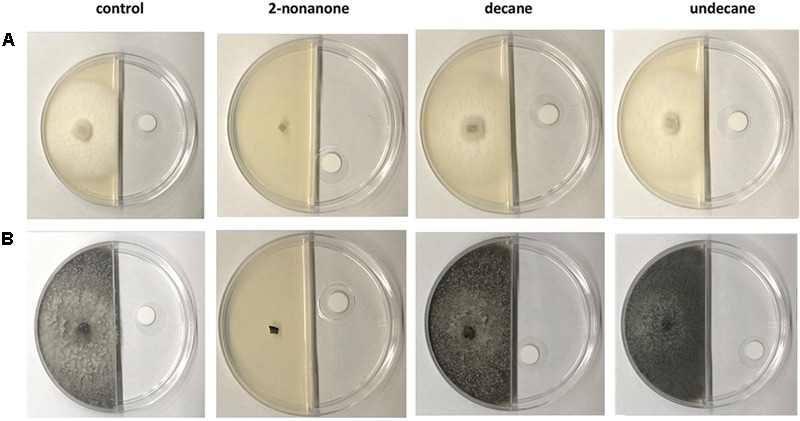
Antifungal bioassay performed on three volatile induced compounds (2-nonanone, decane, undecane) at a concentration of 774 μL/L. 2-nonanone (second column from left) totally inhibited the growth of *E. lata* **(A)** and *B. obtusa* **(B)** after 7 days. No antifungal activity was detected for the other two compounds comparing the fungal growth with the control experiment.

The growth of the fungal species inoculated in the other sector of the Petri dish was monitored, and thus, the influence of the volatile compounds at various saturation levels was assessed. To determine whether the compounds had an antifungal effect, a preliminary experiment with 774 μL/L of 2-nonanone, decane and undecane (expressed as liquid of volatile compound for dish volume) was carried out by allowing the fungal species to grow over 7 days. Under these conditions, the growth of both *B. obtusa* and *E. lata* was completely inhibited, while no effect was observed for the two other induced compounds, decane and undecane (**Figure 7**).

To evaluate the antifungal potency of 2-nonanone, a bioassay was performed by testing five lower concentrations of 2-nonanone: 154.8 μL/L, 77.4 μL/L, 19.3 μL/L, 9.7 μL/L, and 2.4 μL/L. Mycelia growth was further monitored at 3, 5, 7, and 9 days, and the results of inhibition of the mycelium are presented in Supplementary Table S3. At day 9, no mycelium inhibition was observed at a concentration equal or below 19.3 μL/L (for *E. lata*) and 154.8 μL/L (for *B. obtusa*). These results indicate a clear antifungal activity of 2-nonanone against both *E. lata* and *B. obtusa*.

### Main Induction Trends of Volatile and Non-volatile Compounds

With the developed integrated metabolomic approach, various volatile and non-volatile compounds were found to be induced when *B. obtusa* and *E. lata* were co-cultured, and trends regarding their dynamics were highlighted. In the non-volatile fraction, no feature other than *O*-methylmellein, which was very significantly induced, was found to be biosynthesized as a result of the fungal confrontation. In the volatile fractions, several compounds were found to contribute to the metabolomic differentiation between the co-culture and single-culture experiments. Several of these volatiles were discriminant, always detected at all time points, while only two were found to be increasingly produced overtime, as evidenced by the developed multiblock data-mining approach. Interestingly, the induction dynamics of *O*-methylmellein and 2-nonanone were similar, and these compounds were incrementally induced over time in the non-volatile and volatile fractions, respectively.

We demonstrated that 2-nonanone possesses antifungal activity against *B. obtusa* and *E. lata*. *O*-methylmellein is a secondary metabolite that has also been reported to be antifungal. While the activity of *O*-methylmellein was found to be rather selective in inhibiting the development of *B. obtusa* (Glauser et al., 2009), 2-nonanone was shown to be active against both *E. lata* and *B. obtusa*. Based on our metabolomic results, it was not possible to determine whether 2-nonanone was produced by a single strain or both, as it could only be detected in the confrontation experiment and no trace could be recorded in single-culture experiments.

To the best of our knowledge, this study is the first to correlate the induction phenomena at both volatile and non-volatile levels. The obtained results suggest that chemical defense is triggered at these different levels and that the bioactive metabolites identified present similar induction patterns. These findings may indicate that fungi elaborate defense strategies that yield antifungals concomitantly and that such compounds may act additively or synergistically.

Further studies are needed to decipher the various events that trigger these biochemical responses. The proposed analytical and chemometric approach could tackle such issues in an untargeted and unbiased manner.

## Author Contributions

AA, KG, and NL participated in fungal culture preparation. LB, CB, PR, and BS managed the GC-MS experiments and data processing. AA performed the extraction of the non-volatile fractions. AA and P-MA managed the LC-HRMS experiments, data processing and peak-picking. AA, JB, SR, and J-LW participated in the multivariate data analysis. AA, KG, and NL managed the biological evaluation. AA and J-LW managed the project. AA and J-LW conceived the project. All authors participated in manuscript writing and correction.

## Conflict of Interest Statement

The authors declare that the research was conducted in the absence of any commercial or financial relationships that could be construed as a potential conflict of interest.

## Footnotes

1 http://mycoscope.bcis.ch/

2 http://dnp.chemnetbase.com/
