# Supplementary material for: Dynamics of Metabolite Induction in Fungal Co-cultures by Metabolomics at Both Volatile and Non-volatile Levels
Source: Front Microbiol. 2018 Feb 5;9:72. doi: 10.3389/fmicb.2018.00072 (PMC5807337; doi:10.3389/fmicb.2018.00072)

Supplementary Material

Dynamics of metabolite induction in fungal co-cultures by metabolomics at both volatile and non-volatile levels

**Antonio Azzollini^1^**^Δ^**, Lorenzo Boggia^2^**^Δ^**, Julien Boccard^1^, Barbara Sgorbini^2^, Nicole Lecoultre^3^, Pierre-Marie Allard^1^, Patrizia Rubiolo^2^, Serge Rudaz^1^, Katia Gindro^3*^, Carlo Bicchi^2^, Jean-Luc Wolfender^1*^**

**^1^School of Pharmaceutical Sciences, EPGL, University of Geneva, University of Lausanne, CMU - Rue Michel Servet, 1 1211 Geneva 4 – Switzerland**

**^2^Department of Drug Science and Technology, University of Turin, Via P. Giuria 9, 10125, Turin, Italy**

**^3^Agroscope, Plant Protection, Mycology and Biotechnology, Route de Duiller 50, PO Box 1012, 1260 Nyon 1, Switzerland**

^Δ^ Equally contributing first authors.

*** Correspondence:** [jean-luc.wolfender@unige.ch](mailto:jean-luc.wolfender@unige.ch), [katia.gindro@agroscope.admin.ch](mailto:katia.gindro@agroscope.admin.ch)

**Tables S1.** Table S1a and Table S1b show the AMOPLS predictive components (tp1 to to, first row) related to the specific effect of the experimental design (*Culture, Time or Culture×Time*), respectively, for the GC-MS dataset and for the LC-MS dataset (Positive Ionization). The predictive components associated with each specific effect are highlighted.

**Table S1a**

|  | tp1 | tp2 | tp3 | tp4 | tp5 | tp6 | tp7 | tp8 | tp9 | tp10 | tp11 | to |
| --- | --- | --- | --- | --- | --- | --- | --- | --- | --- | --- | --- | --- |
| *Culture* | **91%** | 1% | 2% | **85%** | 4% | 4% | 4% | 4% | 6% | 6% | 6% | 7% |
| *Time* | 3% | **91%** | 7% | 4% | 13% | **56%** | 14% | 13% | **43%** | 21% | 23% | 26% |
| *Culture×Time* | 3% | 3% | **82%** | 5% | **65%** | 17% | **63%** | **64%** | 22% | **45%** | **39%** | 29% |
| *Residuals* | 4% | 4% | 10% | 6% | 18% | 22% | 19% | 19% | 29% | 29% | 32% | 38% |

**Table S1b**

|  | tp1 | tp2 | tp3 | tp4 | tp5 | tp6 | tp7 | tp8 | tp9 | tp10 | tp11 | to |
| --- | --- | --- | --- | --- | --- | --- | --- | --- | --- | --- | --- | --- |
| *Culture* | **81%** | 6% | 8% | **65%** | 14% | 16% | 17% | 19% | 18% | 19% | 21% | 22% |
| *Time* | 5% | **79%** | 7% | 10% | 14% | 16% | **39%** | 19% | **37%** | 19% | 21% | 21% |
| *Culture×Time* | 6% | 7% | **74%** | 11% | **51%** | **44%** | 20% | **34%** | 20% | **33%** | 28% | 25% |
| *Residuals* | 8% | 8% | 11% | 14% | 21% | 23% | 24% | 28% | 25% | 28% | 30% | 31% |

**Table S2.** Steps and parameters used during the peak picking procedure performed using MZmine 2.

| Steps | Parameters | Value | |
| --- | --- | --- | --- |
|  | | PI | NI |
| 1) Raw data methods -> Filtering -> Crop filter | | | |
|  | Retention time (min) | 0.5 to 4.4 | 0.5 to 4.0 |
| 2) Raw data methods -> peak detection Gridmass 2D peak detection | | | |
|  | Minimum height | 1.8E6 | 8E5 |
|  | *m/z* tolerance | 0.01 | 0.01 |
|  | min max width | 0.03 - 0.8 | 0.03 – 0.85 |
|  | smoothing time | 0.10 | 0.05 |
|  | smoothing *m/z* | 0.0500 | 0.500 |
|  | Debugging level: no debug |  |  |
| 3) Peak List methods -> Isotopes -> Isotopic peak grouper | | | |
|  | *m/z* tolerance (ppm) | 5 | 5 |
|  | Retention time tolerance (min) | 0.08 | 0.08 |
|  | Maximum charge | 2 | 2 |
|  | Representative isotope | Lowest *m/z* | Lowest *m/z* |
| 4) Peak List methods -> Alignment -> RNSAC Alignment | | | |
|  | *m/z* tolerance (ppm) | 5 | 5 |
|  | Retention time tolerance (min) | 0.3 | 0.1 |
|  | RT after correction | 0.05 | 0.05 |
|  | Minimum number of points | 20% | 20% |
| 5) Peak List methods-> Filtering -> Duplicate peak filter | | | |
|  | *m/z* tolerance (ppm) | 5 | 5 |
|  | Retention time tolerance (min) | 0.05 | 0.05 |
| 6) Peak List methods-> Gapfilling-> same RT and *m/z* gap filler | | | |
|  | *m/z* tolerance (ppm) | 5 | 5 |
| 7) Removal of the peaks from the blanks and the agar samples from the generated matrix | | | |

**Tables S3.** Estimation of the antifungal potency of 2-nonanone. In Table S3a the mycelium radius (cm) of *E. lata* is reported at different time points (3, 5, 7 and 9 days) and at different concentrations (2.4 µL/L, 9.7 µL/L, 19.3 µL/L, 77.4 µL/L, 154.8 µL/L) of tested compound; the letters _a, _b, _c, _d, _e and _f indicate sample replicates at the different time points. In table S3b the mycelium radius (cm) of *B. obtusa* is reported at the abovementioned time points and concentrations. The label ‘whole sector’ at 7 days and 9 days, indicates that the mycelium covered the whole sector of the Petri dish on which the fungus was inoculated. In such cases the correct measurement of the radius was not possible.

**Table S3a**

|  | Control | 2.4 µL/L | 9.7 µL/L | 19.3 µL/L | 77.4 µL/L | 154.8 µL/L |
| --- | --- | --- | --- | --- | --- | --- |
| 3day_E. lata_a | 0.5 | 0.4 | 0.3 | 0.2 | 0 | 0 |
| 3day_E. lata_b | 0.45 | 0.35 | 0.3 | 0.2 | 0 | 0 |
| 3day_E. lata_c | 0.5 | 0.4 | 0.35 | 0.3 | 0 | 0 |
| 3day_E. lata_d | 0.45 | 0.35 | 0.3 | 0.3 | 0 | 0 |
| 3day_E. lata_e | 0.5 | 0.4 | 0.4 | 0.2 | 0 | 0 |
| 3day_E. lata_f | 0.5 | 0.4 | 0.3 | 0.25 | 0 | 0 |
| 5day_E. lata_a | 0.9 | 0.5 | 0.55 | 0.4 | 0.3 | 0.2 |
| 5day_E. lata_b | 0.9 | 0.5 | 0.5 | 0.45 | 0.25 | 0.2 |
| 5day_E. lata_c | 0.95 | 0.6 | 0.5 | 0.4 | 0.3 | 0.25 |
| 5day_E. lata_d | 0.95 | 0.55 | 0.5 | 0.5 | 0.2 | 0.3 |
| 5day_E. lata_e | 0.9 | 0.6 | 0.5 | 0.4 | 0.3 | 0.2 |
| 5day_E. lata_f | 1 | 0.6 | 0.6 | 0.4 | 0.2 | 0.2 |
| 7day_E. lata_a | 2.5 | 2.4 | 2.6 | 2.35 | 1.5 | 1.5 |
| 7day_E. lata_b | 2.4 | 2.6 | 2.7 | 2.65 | 1.9 | 1.5 |
| 7day_E. lata_c | 2.3 | 2.7 | 2.5 | 2.4 | 1.8 | 1.7 |
| 7day_E. lata_d | 2.7 | 2.3 | 2.3 | 2.6 | 2 | 2 |
| 7day_E. lata_e | 2.6 | 2.5 | 2.4 | 2.5 | 1.9 | 1.5 |
| 7day_E. lata_f | 2.5 | 2.5 | 2.5 | 2.5 | 1.8 | 1.5 |
| 9day_E. lata_a | 3.5 | 3 | 3 | 3.5 | 2.5 | 2.5 |
| 9day_E. lata_b | 3.5 | 3.4 | 3.5 | 3 | 3 | 2.5 |
| 9day_E. lata_c | 3.5 | 3.5 | 3.4 | 3.4 | 3 | 2.5 |
| 9day_E. lata_d | 3.4 | 3.5 | 3.2 | 3.2 | 2.8 | 2.5 |
| 9day_E. lata_e | 3.2 | 3.2 | 3.5 | 3.5 | 2.7 | 2.6 |
| 9day_E. lata_f | 3 | 3.5 | 3.5 | 3.5 | 3 | 3 |

**Table S3b**

|  | Control | 2.4 µL/L | 9.7 µL/L | 19.3 µL/L | 77.4 µL/L | 154.8 µL/L |
| --- | --- | --- | --- | --- | --- | --- |
| 3day_B.obtusa_a | 2 | 1.7 | 1.4 | 1 | 0.1 | 0 |
| 3day_B.obtusa_b | 2.2 | 1.5 | 1.5 | 1 | 0.15 | 0 |
| 3day_B.obtusa_c | 1.8 | 1.7 | 1.3 | 0.9 | 0 | 0 |
| 3day_B.obtusa_d | 2.1 | 1.7 | 1.3 | 1.1 | 0.1 | 0 |
| 3day_B.obtusa_e | 1.9 | 1.7 | 1.4 | 1 | 0.2 | 0 |
| 3day_B.obtusa_f | 2 | 1.6 | 1.4 | 1 | 0.05 | 0 |
| 5day_B.obtusa_a | 4 | 3.3 | 3.2 | 3 | 1.5 | 1.1 |
| 5day_B.obtusa_b | 3.9 | 3.2 | 3.2 | 3 | 1.5 | 1.1 |
| 5day_B.obtusa_c | 4 | 3.4 | 3.2 | 2.9 | 1.1 | 1.2 |
| 5day_B.obtusa_d | 4 | 3.4 | 3.4 | 3 | 1.4 | 1.2 |
| 5day_B.obtusa_e | 4 | 3.4 | 3.2 | 2.8 | 1.25 | 1.1 |
| 5day_B.obtusa_f | 4 | 3.4 | 3.3 | 2.8 | 1.25 | 1.5 |
| 7day_B.obtusa_a | Whole sector | Whole sector | Whole sector | Whole sector | Whole sector | Whole sector |
| 7day_B.obtusa_b | Whole sector | Whole sector | Whole sector | Whole sector | Whole sector | Whole sector |
| 7day_B.obtusa_c | Whole sector | Whole sector | Whole sector | Whole sector | Whole sector | Whole sector |
| 7day_B.obtusa_d | Whole sector | Whole sector | Whole sector | Whole sector | Whole sector | Whole sector |
| 7day_B.obtusa_e | Whole sector | Whole sector | Whole sector | Whole sector | Whole sector | Whole sector |
| 7day_B.obtusa_f | Whole sector | Whole sector | Whole sector | Whole sector | Whole sector | Whole sector |
| 9day_B.obtusa_a | Whole sector | Whole sector | Whole sector | Whole sector | Whole sector | Whole sector |
| 9day_B.obtusa_b | Whole sector | Whole sector | Whole sector | Whole sector | Whole sector | Whole sector |
| 9day_B.obtusa_c | Whole sector | Whole sector | Whole sector | Whole sector | Whole sector | Whole sector |
| 9day_B.obtusa_d | Whole sector | Whole sector | Whole sector | Whole sector | Whole sector | Whole sector |
| 9day_B.obtusa_e | Whole sector | Whole sector | Whole sector | Whole sector | Whole sector | Whole sector |
| 9day_B.obtusa_f | Whole sector | Whole sector | Whole sector | Whole sector | Whole sector | Whole sector |

**Supplementary Figure S1.** Heat map reporting compound abundances in samples cultivated in different agar volumes. Every column represents a different sample. Every row represents a compound for which the maximum peak area is reported in red and the minimum in blue. On the x-axis, the codes read as follows: for example, 2 ml_Bo_a represents a 2 mL volume of PDA on which a single culture of *B. obtusa* (or, in the case of Eu and BoxEu, a single culture of *E. lata* and a co-culture, respectively) is grown; the letters _a, _b, and _c indicate the respective replicates of the single culture and the co-cultures.


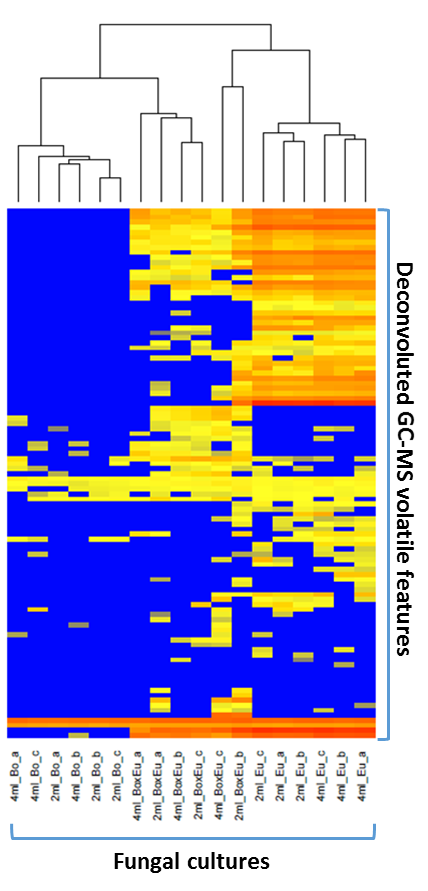


**Supplementary Figure S2.** Electronic Impact-Mass Spectrum of the sesqui@RT 34.879 compound.


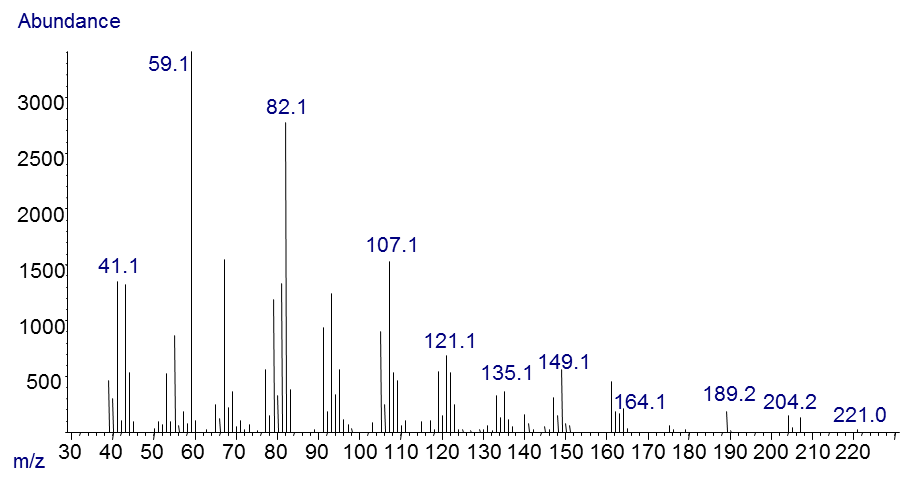


**Supplementary Figure S3.** Electronic Impact-Mass Spectrum of the sesqui@RT 28.691 compound.


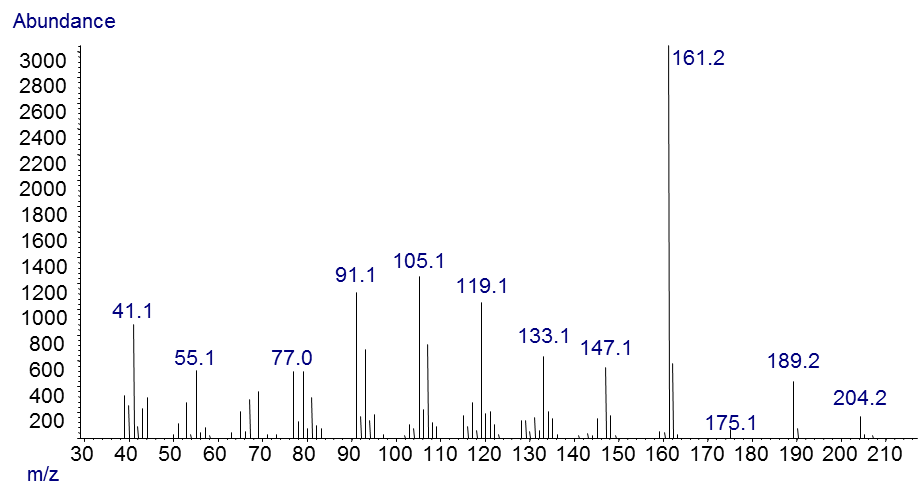


**Supplementary Figure S4.** EI-MS fragmentation patterns of the 2-nonanone standard compound and the same compound detected in *E. lata* and *B. obtusa* co-cultures.


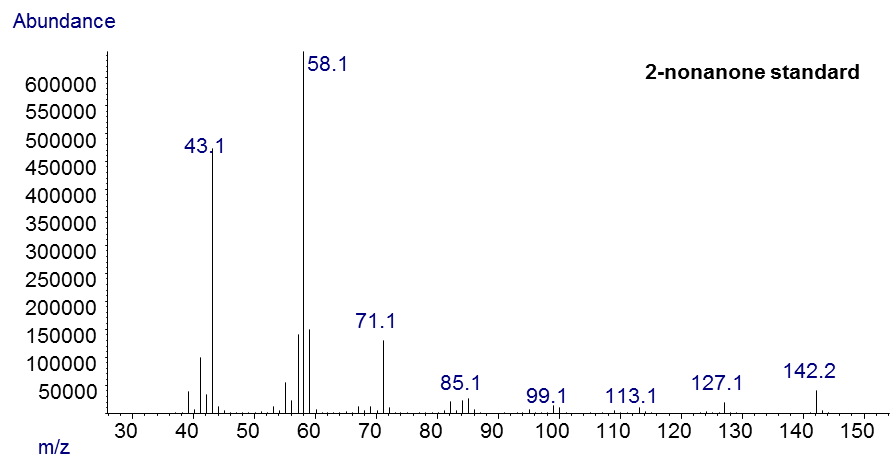


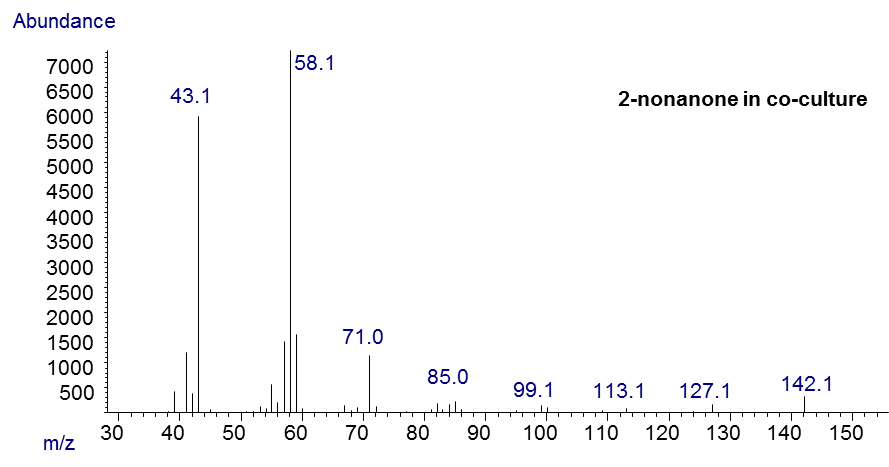


**Supplementary Figure S5.** Extracted-ion chromatogram (XIC) for the ion [193.09@RT](mailto:193.09@RT)1.44 (*O*-methymellein). Red trace= co-culture; blue trace= *Botryosphaeria obtusa*; green trace= *Eutypa lata* on day 9.


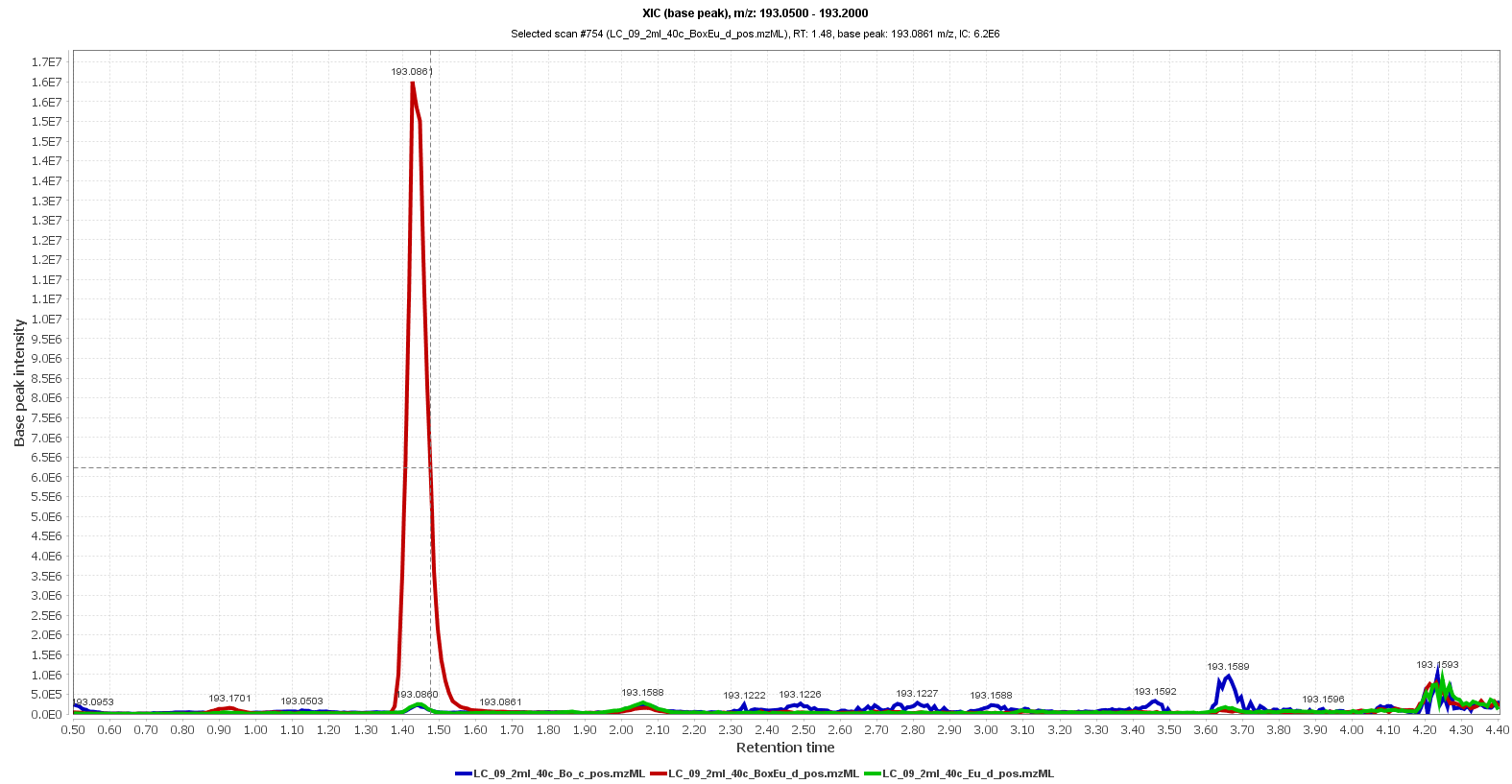

Supplement: Supplementary file 1 [file Data_Sheet_1.docx]
